# The roles of banana peel powders to alter technological functionality, sensory and nutritional quality of chicken sausage

**DOI:** 10.1002/fsn3.1847

**Published:** 2020-08-31

**Authors:** Hana Binti Mohd Zaini, Mohd Dona Bin Sintang, Wolyna Pindi

**Affiliations:** ^1^ Faculty of Food Science and Nutrition Universiti Malaysia Sabah Kota Kinabalu Sabah Malaysia

**Keywords:** banana peel, dietary fiber, functional food, rheology, sausage

## Abstract

Chicken sausages included with three different quantities of banana (*Musa balbisiana*) peel powder. The technological properties (cooking yield, texture, water‐holding capacity, color, rheology, and texture), composition, and sensory acceptability were assessed. In storage study, lipid oxidation of the best formulation from the sensory score was evaluated. The inclusion of banana peel powder (BPP) raises the nutritional value with regard to an increase in dietary fiber and a reduction in the sausage fat content. The addition of BPP also causes a significant increase in the cooking yield and water‐holding capacity. Additionally, storage modulus values increase with the increase in the BPP's concentration. However, with BPP incorporation, a hard texture and darkening of the sausage were observed. Interestingly, our findings exhibit the compromise in microstructural of chicken sausage with high percentage of BPP manifested by the high storage modulus and hardness but with low resistance toward stress, short linear viscoelastic region. This aspect also caused a significant change in the sensory score. The TBA value in the sausage containing 2% BPP exhibited a delay in lipid oxidation up to 55%, prompting its antioxidant potential. Generally, the incorporation of BPP to chicken sausage changes its properties. BPP has been a potential candidate as a value‐adding ingredient that may be used during meat preparation since it positively influences the nutritional value and specific technological properties of the food.

## Graphical abstract

The inclusion of banana peel powder (BPP) raises the nutritional value with regard to an increase in dietary fiber and a reduction in the sausage fat content. The addition of BPP also causes a significant increase in the cooking yield and water‐holding capacity. The incorporation of BPP to chicken sausage may change its properties. BPP has been a potential candidate as a value‐adding ingredient that may be used during meat preparation since it positively influences the nutritional value and specific technological properties of the food.

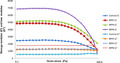

## 1 INTRODUCTION

Foods like meat, which have an animal origin, are typically a great source of protein, vitamins, essential fatty acids, and minerals required by the human body for good health and wellbeing. Changes like population growth, urbanization, economic, and market expansion create a higher demand for meat products. The global per capita meat consumption (average) has risen from 20 kg in 1961 to 43 kg in 2014 (Ritchie and Roser, 2017).

In tropical and subtropical areas, banana is among the most cultivated and consumed food. Data indicated that global banana output reached a record high of 114 million tonnes in 2017 with a compounded annual growth rate of 3.2% (FAO, 2017). Banana peel (BP) constitutes approximately 38% of the total weight of the banana (González‐Montelongo, Gloria, & González, 2010), which indicates that banana peels weighing approximately 40 million tons were generated annually. BP is typically discarded as waste, which is an environmental problem and causes industrial concern.

BP contains bioactive chemicals such as phlobatannins, tannins, flavonoids, alkaloids, glycosides, terpenoids, and anthocyanins, which may be used for their specific biological and pharmacological aspects like antibacterial, antidiabetic, antihypertensive, and anti‐inflammatory characteristics (Pereira & Maraschin, 2015). Additionally Rebello et al. (2014) and Sundaram, Anjum, Dwivedi, & Rai (2011) mentioned that BP is rich in micronutrients and antioxidants like polyphenols, prodelphinidins, carotenoids, and catecholamines. Rattanvichai & Cheng (2015) reported that the bioactive chemicals present in BP provide useful medicinal properties, including immunostimulant effects, to the fruit peel. Additionally, BP has a high content of vitamins, dietary fibers, proteins, potassium, and polyunsaturated fatty acids (Emaga, Andrianaivo, Wathelet, Tchango, & Paquot, 2007). Therefore, BP is one of those waste products which may potentially be used as a functional additive in the food industry.

Dietary fiber (DF) is a nonstarch polysaccharide having resistance to absorption and digestion by the enzymes present in the human gastrointestinal tract (Trowell, 1978). Since the human body cannot digest and absorb dietary fiber, moisture absorption in the digestive system is affected (Yang, Ma, Wang, & Zheng, 2017). It may raise the food volume in the gut, providing a sense of satiety, therefore preventing overconsumption of food (Manzoni, Castelnuovo, and Molinari, 2008). Additionally, DF facilitates gastrointestinal peristalsis, thereby preventing constipation (Tse, Leung, Chan, Sien, & Chan, 2000) and also absorbs toxins or harmful substances in the gut (Borycka, 2010). Also, DF has been demonstrated to reduce postprandial blood glucose level, insulin, glucose, and triglyceride concentration (Ma & Mu, 2016), while also lowering blood cholesterol levels (Villanueva‐Suárez, Pérez‐Cózar, Mateos‐Aparicio, & Redondo‐Cuenca, 2016).

However, the effort to increase fiber consumption in the diet is a difficult challenge. For this reason, fiber should not only afford health benefits but also provide enhanced technological properties to encourage continued high‐fiber product intake. Fiber is ideal for meat product development due to its water retention property, neutral flavor, and reduces cooking loss. DF has diverse functional properties such as gel‐forming ability, water‐binding capacity, oil adsorption capacity, and organic and mineral‐binding capacity, which may improve the meat product quality and characteristics (Biswas, Kumar, Bhosle, Sahoo, & Chatli, 2011). Several studies showed that the incorporation of fibers (e.g., sugar beet fiber, potato starch, oat fiber) in meat emulsion promotes water retention capacity, resulting in a low cooking loss.

Consumers are growing increasingly concerned about the importance of consuming a healthy diet, which is resulting in an increasing demand for healthy foods, including meat. Though meat products are a primary source of several nutrients, they have also been associated with adverse health effects like diabetes, high cholesterol, obesity, cardiovascular, and other diseases (Grasso, Brunton, Lyng, Lalor, & Monahan, 2014; Jiang & Xiong, 2016; Mora‐Gallego, Guardia, Serra, Gou, & Arnau, 2016). Therefore, there are efforts to produce “healthier meat products” by reducing unhealthy components like saturated fats, salt, and nitrates (Grasso et al., 2014; Mora‐Gallego et al., 2016). Another technique in this direction is the addition of health‐promoting bioactive ingredients like dietary fibers in meat (Grasso et al., 2014; Resconi et al., 2016), along with other substances like natural antioxidants and probiotics (Grasso et al., 2014; Horita et al., 2016; Jiang & Xiong, 2016) and so on.

To date, evidence on the association between the lack of vegetable intake and low intake of dietary fiber is well documented by several researchers (Ellis, Zickgraf, Galloway, Essayli, & Whited, 2018; Tiemeier, Tharner, Hofman, & Hoek, 2017). A similar effect would be caused by a low intake of whole‐grain products in picky eaters (Ellis et al., 2018; Tiemeier et al., 2017). According to NCCFN (NCCFN, 2017), in accordance with WHO, one should consume at least 30 g of fiber a day regardless of gender; however, most people worldwide consumed less than the recommended amount. The factor may be due to the rapid urbanization with a variety of food sources has changed current food intake as the people are likely to have poor eating habits by consuming high‐cholesterol foods, processed or fast foods, high‐calorie snacks, excessive intake of calorie, having irregular mealtime and skipping breakfast, and eating an unbalanced diet (Nemnunhoi & Sonika, 2016). Thus, there is a need for incorporating DF in highly consumed food such as meat product to enhance DF intake without drastically change the eating habit.

Several solutions have been explored to augment meat products with several fiber sources (Mehta, Ahlawat, Sharma, & Dabur, 2015). Apart from producing meat products with the desired nutritional values, fiber‐enriched meat products provide distinct advantages like better technological characteristics like higher binding capacity toward water or oil, in addition to providing antioxidant properties (Sáyago‐Ayerdi, Brenes, & Goñi, 2009).

Considering these reasons, a study on the utilization of banana peel for enriching meat products with fibers was conducted. The “Waste‐to‐Wealth” concept has been used in the context of this study.

## 2 MATERIALS AND METHODS

### 2.1 Preparation of banana peel powder (BPP)

Ripe *Musa balbisiana* (stage 7 of ripening; Fernandes and Bonaldo, 2011) were bought locally from the market around Kota Kinabalu, Sabah, Malaysia. The bananas were washed using tap water to get rid of any debris, post which the peel and pulp were separated. The peels were sliced into 2 cm by 4 cm pieces and treated with 0.5% (w/v) citric acid solution to halt enzymatic browning. The solution was then drained, and the sliced peels were dried using a drying cabinet (Thermolite, Malaysia) for 48 hr at 40°C (Agama‐Acevedo, Saňudo‐Barajas, Vélez De la Rocha, González‐Aguilar, & Bello‐Pérez, 2009). The peels were subsequently grounded and screened using 60 mesh screens (250 µm).

### 2.2 Chicken sausage preparations

The Desa Hatchery Sdn. Bhd., Lok Kawi, Sabah, Malaysia, was selected for the purchase of boneless chicken breast. The sausage preparation was done as suggested by Syuhairah, Huda, Syahariza, & Fazilah (2016), including some modifications. Sausage constituents (chicken breast, fat, ice water, potato starch, isolated soy protein, sugar, pepper, and salt) were well mixed by using a cutter to get a homogenous mixture, which was mixed with BPP at three different constitutions (2%, 4%, and 6% dry matter). A sausage stuffer was used to fill the cellulose casing with meat batter. The specimens were labeled and steamed for 30 min at a temperature of 75 ± 2°C. The sausages were then dipped in cold water for 20 min at a temperature of 15°C. Subsequently, the water was drained, and the sausages were maintained at 4°C inside airtight bags.

### 2.3 Proximate analysis

The methods, as described by AOAC (2000), were employed to get the compositional properties of these samples. The **oven‐drying** method (950.4B) was used to find the moisture content. The determination of protein content was done using the Kjeldahl method (981.10) using an automatic Kjeldahl nitrogen analyzer (Kjeltec® 2300 Analyzer Unit, Foss Tecator AB). The fat content (method 960.69, ether extractable component) was ascertained using the Soxhlet method by employing a solvent extraction system (Soxtec® Avanti 2050 Auto System, Foss Tecator AB, Sweden). Ash content was ascertained by using a muffle furnace overnight at 550°C (923.153).

### 2.4 Total dietary fiber (TDF) analysis

The enzymatic–gravimetric analysis, as suggested in AOAC (2005), was used to ascertain the TDF. A gram defatted dried sample was measured and was subjected to digestion. The sample, along with ɑ‐amylase, was initially incubated at 98–100°C for 15 min. After that, the incubation process included protease and amyloglucosidase, respectively, for 30 min, at 60°C.

Acetone and 95% ethanol were used to filter and remove the residue, which was then dried, and its weight was measured. One similar sample was used to determine the protein content, while another similar sample was subjected to 525°C in a muffle furnace to ascertain the ash content. DF value was calculated as the residue weight less the weight of protein and ash.

### 2.5 Water‐holding capacity

The centrifugation technique with some modifications was used to determine the water‐holding capacity (Zhuang, Nelson, Trablesi, & Savage, 2007). A 10‐g sample was mixed with 15 ml, 0.6 M NaCl solution inside a tube, which was then centrifuged at 4°C at a 3,000 *g* speed for 15 min. The water‐holding capacity (WHC) is ascertained as follows:

$$\text{WHC}\left(\%\right)=\frac{\text{Weight after centrifuge}‐\text{Weight before centrifuge}}{\text{Weight before centrifuge}}\times 100\%$$

### 2.6 Cooking yield

The cooking yield was ascertained using the weight of the sample for every treatment and weight difference in the sausages before and after precooking using steam at 75°C for 30 min (Choi et al., 2012). The cooking yield is determined as follows:

$$\text{Cooking yield}\left(\%\right)=\frac{\text{Initial weight of sample}‐\text{Final weight of sample}}{\text{Initial weight of sample}}\times 100\%$$

### 2.7 Color

The color of the samples was determined using a Konica Minolta chromameter (CR‐400, Chroma meter, Japan; Choe et al., 2018). Six measurements were taken perpendicularly, where the different surfaces of the sausage were photographed. The values were recorded as CIE L* (lightness), a* (redness), and b* (yellowness).

### 2.8 Texture profile analysis (TPA)

Texture analysis was conducted using TA.XT Plus Texture Analyzer (Stable Micro System, UK; Hu et al., 2016). For this analysis, each sample had six replicates. The samples were uniform with a 20 mm height and 25 mm diameter and compression to 50% of their actual size. The attributes recorded during this analysis are cohesiveness, springiness, hardness, and chewiness.

### 2.9 Dynamic rheology

Rheological aspects were assessed using a rheometer (AR500 TA Co. Ltd., UK) with the following setup: testing temperature set at 10°C in a stainless‐steel cone plate with a diameter of 60 mm and a gap width of 2 mm, and a sweep stress test from 0.1 to 100 Pa, and at 1 Hz frequency. Sample analysis was done in triplicate.

### 2.10 Sensory evaluation

Sausages were cooked in four formulations for sensory assessment. This evaluation used a seven‐point hedonic scale (1 = extremely dislike, 7 = extremely like). The sausages were cut uniformly and offered in random order. Forty untrained students of the faculty were chosen to form the sensory panelist set. Taste, aroma, appearance, hardness, color, juiciness, and overall acceptability were the parameters being assessed in the test.

### 2.11 Lipid oxidation

For the lipid oxidation study, the best formulation from the sensory evaluation was chosen and compared with the control sample. The 2‐thiobarbituric acid (TBA) assay was carried out according to the procedure of Oussalah, Caillet, Salmieri, Saucier, & Lacroix (2006). Sausage sample (5 g) was mixed with 20 ml of trichloroacetic acid and homogenized in a bag mixer for 2 min. After filtration, 2 ml of TBA solution was added in the 2 ml of the filtrate. The test tubes contain filtrate and TBA were incubated at 95°C for 35 min; then, the absorbance was measured at 532 nm by using a spectrophotometer (PerkinElmer, USA). TBA value was expressed as mg malonaldehyde/kg of sausage.

### 2.12 Statistical analysis

The one‐way ANOVA test was used to evaluate the effects of using BPP on the sensory and physicochemical properties of chicken sausage. SPSS version 24.0 statistical processor software was used to analyze the data. The level of significance among the means for the several attributes was set as *p* < .05.

## 3 RESULTS

### 3.1 Proximate composition and total dietary fiber content

The banana peel powder prepared in this study comprised 11.11% in moisture, 4.85% protein, 9% ash, 7.06% fat, and 44.03% of total dietary fiber. The total dietary fiber and the sausages mixed with different percentages of banana peels are mentioned in Table 1. Dietary fiber has a noteworthy ability to hold water, but for the samples of this study, the moisture content reduced as the BPP addition was increased. This could probably happen because of the moisture content of BPP (11.111%) which is lesser than the moisture content in chicken meat. Additionally, the inclusion of dietary fiber, even in its dry form, also causes a reduction of moisture in the treated samples (Yadav, Pathera, Islam, Malik, & Sharma, 2018). Similar observations were reported by Talukder & Sharma (2010), who found that the inclusion of wheat bran to chicken patty led to a decrease in moisture content.

**Table 1:** Proximate composition and total dietary fiber of chicken sausage with different percentage of banana peel powder

Composition	Mean ± *SD*			
	BPP0	BPP2	BPP4	BPP6
Moisture (%)	70.62 ± 0.34^a^	70.12 ± 2.29^a^	67.96 ± 0.77^bc^	66.94 ± 0.43^c^
Ash (%)	3.04 ± 1.20^c^	3.98 ± 1.72^bc^	5.13 ± 0.81^ab^	5.77 ± 0.01^a^
Protein (%)	13.73 ± 0.29^a^	12.70 ± 0.62^b^	9.87 ± 0.43^c^	3.39 ± 0.16^d^
Fat (%)	9.18 ± 0.75^a^	7.67 ± 0.29^b^	5.63 ± 1.02^c^	4.58 ± 0.36^c^
TDF (%)	0.83 ± 0.70^b^	1.70 ± 0.99^a^	NA	NA

Note

^a‐c^Mean ± *SD* in same row with different superscript indicates that there are significant different (*p* < .05).

Abbreviations: BPP0, control (without banana peels powder); BPP2, added 2 percent banana peel powder; BPP4, added 4 percent banana peels powder; BPP6, added 6 percent banana peel powder; NA, not available.

Protein and fat present in the treated samples also reduced significantly (*p* < .05) because of the lesser fat and protein content present in BPP. Earlier studies like the inclusion of oat flour in chicken nuggets (Santhi & Kalaikannan, 2014) and the addition of carrageenan flour to meat kofta (Modi, Yashoda, & Naveen, 2008) bear similar results.

A significant increase (*p* < .05) was observed for ash content as more BPP was added to the sausage. Higher ash presence could probably be caused by the increasing concentration of total dietary fibers, minerals, and resistant starch present in the BPP (Pereira & Maraschin, 2015). Mineral and vitamin content affect the ash content. It may, therefore, be concluded that the inclusion of BPP provides for a higher nutritional value considering vitamin and mineral content (Park, Kim, Park, Han, & Kim, 2011). The study conducted by López‐Vargas, Fernández‐López, Pérez‐Álvarez, & Viuda‐Martoz (2014) indicated similar findings where the ash content in pork burgers was found to increase as the added passion fruit albedo was increased (2.5%–5%).

The control samples were observed to have a significantly lower TDF content (*p* < .05) when compared to that of the BPP‐augmented sausage. A high level of TDF in BPP could potentially be the reason behind the increase of TDF in the treated sample. As the TDF in BPP2 started to exhibit a substantial increase, the TDF is expected to be directly proportional to the BPP in the sausage. Similar conclusions were drawn (Ammar, 2017) in the case of chicken nuggets, where the TDF increased significantly upon the addition of 5%–10% orange albedo. A study by Huang, Tsai, & Chen (2011) demonstrated that the inclusion of wheat, oats, and inulin at 3% and 7%, respectively, caused an increase in the dietary fiber of the sausages (2.86%–5.91%).

### 3.2 Water‐holding capacity and cooking yield

Mahmoud, Abou‐Arab, & Abu‐Salem (2017) comment that the water‐holding capacity of meat may be understood as its ability to retain its water content even with external pressure (gravity and temperature) applied to it. This aspect is essential in meat products since water‐holding capacity influences the cooking loss and sensory quality of the meat (Aleson‐Carbonell, Fernandez‐Lopez, Perez‐Alvarez, & Kuri, 2005).

The cooking yield and water‐holding capacity of sausages with added BP powder are specified in Table 2. WHC gives information regarding the ability of the product to retain water, and therefore impacts the physical properties of the product (Zaini, Sintang, Wahab, Hamid, & Pindi, 2019). These include texture aspects, cooking loss, and mouth feel. Water loss is a widespread issue faced by this industry because not only does it reduce product weight but it also causes liquid accumulation on the surface (Hautrive, Oliveira, Silva, Terra, & Campagnol, 2008). As specified in Table 2, the WHC of the treated sample increases with the addition of the banana peel powder, and it is observed to have a significant increase (*p* < .05) at 6% BPP addition level.

**Table 2:** Water‐holding capacity and cooking yield of chicken sausage with different percentage of banana peel powder

Treatments	Mean ± *SD*	
	Water‐holding capacity (%)	Cooking yield (%)
BPP0	6.54 ± 1.08^b^	96.96 ± 0.75^b^
BPP2	6.67 ± 0.66^b^	98.69 ± 0.37^a^
BPP4	7.97 ± 0.64^ab^	99.26 ± 0.49^a^
BPP6	9.28 ± 0.33^a^	99.54 ± 0.28^a^

Note

^a,b^Mean ± *SD* in same column with different superscript indicates that there are significant different (*p* < .05).

Abbreviations: BPP0, control (without banana peels powder); BPP2, added 2 percent banana peel powder; BPP4, added 4 percent banana peels powder; BPP6, added 6 percent banana peel powder.

The WHC increase may be explained by the dietary fiber content of BPP. Since dietary fiber is hydrated, the pore space in fiber particles is occupied by water molecules. Additionally, the observed increase of WHC in the treated sample may be due to the high temperature‐induced gelatinization of hydrated banana peel start (Ali, El‐Anany, & Gaafar, 2011).

Similar results were reported by Ammar (2017) where the addition of 5% albedo orange to sausage resulted in a rise of WHC up to 75.68%, whereas the control sausage had a WHC of 73.61%.

Table 2 also highlights that cooking yield for a BPP‐augmented sample is more than that of the control sample (*p* < .05). Still, there is no marked incremental delta between the samples treated with different BPP percentage (*p* < .05).

Heating the fiber causes swelling and gelatinization. The number of polysaccharides present in the dietary fiber determines these processes (Sánchez‐Alonso, Haji‐Maleki, & Borderias, 2006). Several constituents like starch and pectin interact with the protein and lead to a reduced ability for moisture migration during cooking (Garcia‐Garcia & Totosaus, 2008), which results in the reduction of cooking loss.

### 3.3 Color

Adding BPP to chicken sausages affected the color, and the data are specified in Table 3. The incorporation of BPP causes the sausages to have a darker color (*p* < .05). Color affects consumer acceptance of the product. As per Pietrasik & Duda (2000), the color of the products belonging to the frankfurter type was typically affected because of the amount of water and fat content, given myoglobin was kept constant. The results of the study are in agreement with the abovementioned statement, suggesting that including BPP in the sausages causes lightness. For meat batter and sausage, the lightness reduced significantly (*p* < .05) due to the fact that the fiber of the dry banana peel had a typical brownish color which was potentially transferred to the studied samples, thereby causing a change in the sausage and the batter. It is also in agreement with the study by Bastos et al. (2014), where it was reported that precooked browning might be due to nonenzymatic reaction among the amino acids in meat and the carbohydrates in BPP.

**Table 3:** Color parameter of chicken sausage with different percentage of banana peel powder

Treatment	L*	a*	b*
BPP0	73.48 ± 0.29^a^	1.13 ± 0.76^d^	13.40 ± 0.14^a^
BPP2	60.32 ± 0.32^b^	1.36 ± 0.58^c^	9.48 ± 0.28^b^
BPP4	53.13 ± 0.56^c^	2.18 ± 0.17^ab^	9.31 ± 0.31^b^
BPP6	49.72 ± 0.34^d^	2.36 ± 0.17^ab^	9.25 ± 0.48^b^

Note

^a‐d^Mean ± *SD* in same column with different superscript indicates that there are significant different (*p* < .05).

Abbreviations: BPP0, control (without banana peels powder); BPP2, added 2 percent banana peel powder; BPP4, added 4 percent banana peels powder; BPP6, added 6 percent banana peel powder.

The redness parameter (a*) indicated a substantial difference between the treated samples and the control sample (*p* < .05), with its value increasing with a further addition of BPP in the sausages. The addition of 2% BPP caused the redness value to increase by up to 20% as compared to the control group. Bastos et al. (2014) concluded that adding more fiber as an ingredient leads to an increase in the redness parameter. The yellowness attribute (b*) reduced significantly (*p* < .05) when compared to the control samples without any significant difference between the tested samples. The decrease in yellowness (b*) shows that BPP has a strong water‐holding or retaining capacity (Aleson‐Carbonell et al., 2005). The results have been in line with those reported by Hughes, Cofrades, & Troy (1997), where a decrease in the fat level reduced the yellowness and lightness but raised the redness of frankfurters.

### 3.4 Texture

The changes in the textural aspect of the sausages with different BPP levels are provided in Table 4. The chewiness, hardness, and cohesiveness have a noteworthy effect after BPP addition (*p* < .05). However, no significant difference was detected in terms of springiness (*p* > .05). The control sample was determined to have more hardness compared to other treatments (*p* < .05). Nonmeat protein presence leads to a better protein network, which has a better resistance against compression (Yousef & Barbut, 2011). This may be responsible for the hard sausage texture when prepared with banana peel powder. Makri & Douvi (2014) also reported results in line with the abovementioned, where the inclusion of 7.5% and 10% corn starch caused a significant rise in the hardness of fish patties (*p* < .05).

**Table 4:** Texture of chicken sausage with different percentage of banana peel powder

Treatment	Hardness (*N*)	Cohesiveness	Springeness (mm)	Chewiness (*N* x mm)
BPP0	46.60 ± 10.60^d^	0.64 ± 0.56^a^	0.87 ± 0.03^a^	18.07 ± 4.06^c^
BPP2	60.20 ± 1.49^c^	0.60 ± 0.05^ab^	0.85 ± 0.02^a^	28.43 ± 2.56^b^
BPP4	60.98 ± 0.55^c^	0.54 ± 0.05^bc^	0.86 ± 0.01^a^	30.57 ± 2.80^b^
BPP6	84.99 ± 3.17^a^	0.47 ± 0.04^c^	0.82 ± 0.03^a^	46.65 ± 1.71^a^

Note

^a‐d^Mean ± *SD* in same column with different superscript indicates that there are significant different (*p* < .05).

Abbreviations: BPP0, control (without banana peels powder); BPP2, added 2 percent banana peel powder; BPP4, added 4 percent banana peels powder; BPP6, added 6 percent banana peel powder.

Several studies indicate that adding rice bran fiber, wheat fiber, kimchi fiber, and fiber from other potential sources has been established to increase the hardness of meat products (Choi et al., 2007; Cofrades, Guerra, Carballo, Fernandes‐Martin, & Colmenero, 2000). Contrarily, several studies have indicated that the inclusion of dietary fiber caused higher elasticity and reduced the hardness of meat products. For instance, the study conducted by Garcia, Caceres, & Selgas (2002) mentioned that using fruit fiber with sausage caused a decrease in the hardness score. Several factors could be responsible for the multiple effects of dietary fiber on meat product texture. Some of the factors could be the processing method (fresh/dried), fat type (soluble/insoluble), and the percentage of meat (Yadav et al., 2018).

Cohesiveness refers to the strength of the molecular bonds inside a food product (Sarıçoban, Yılmaz, & Karakaya, 2009). The result agrees with the results from the study conducted by Viuda‐Martos, Ruiz‐Navajas, Fernandez‐Lopez, & Perez‐Alvarez (2010), where the addition of 1% orange fiber reduced the cohesiveness of the meatballs (Sanchez‐Zapata et al., 2010). A decrease in cohesiveness was also observed when 5%, 10%, and 15% tiger bean fiber was added to pork patties. The observed reduction in cohesiveness may be caused by a reduction of the fat content, as described in Table 1, when a higher amount of BPP is added. The result is supported by the conclusions from the studies of Ktari, Smaoui, Trabelsi, Nasri, & Ben Salah (2014), which claim that fiber elimination and fat addition to the meat led to an increase in cohesiveness.

The chewiness value for the control samples was lesser than that of the control samples (*p* < .05). As per Barretto, Pollonio, & Pascheco (2014), higher energy is spent to chew fiber‐rich food. Additionally, hardness also influences the chewiness, where chewiness increases with an increase in texture hardness (Huang et al., 2011).

### 3.5 Dynamic rheology

**Figure 1:**
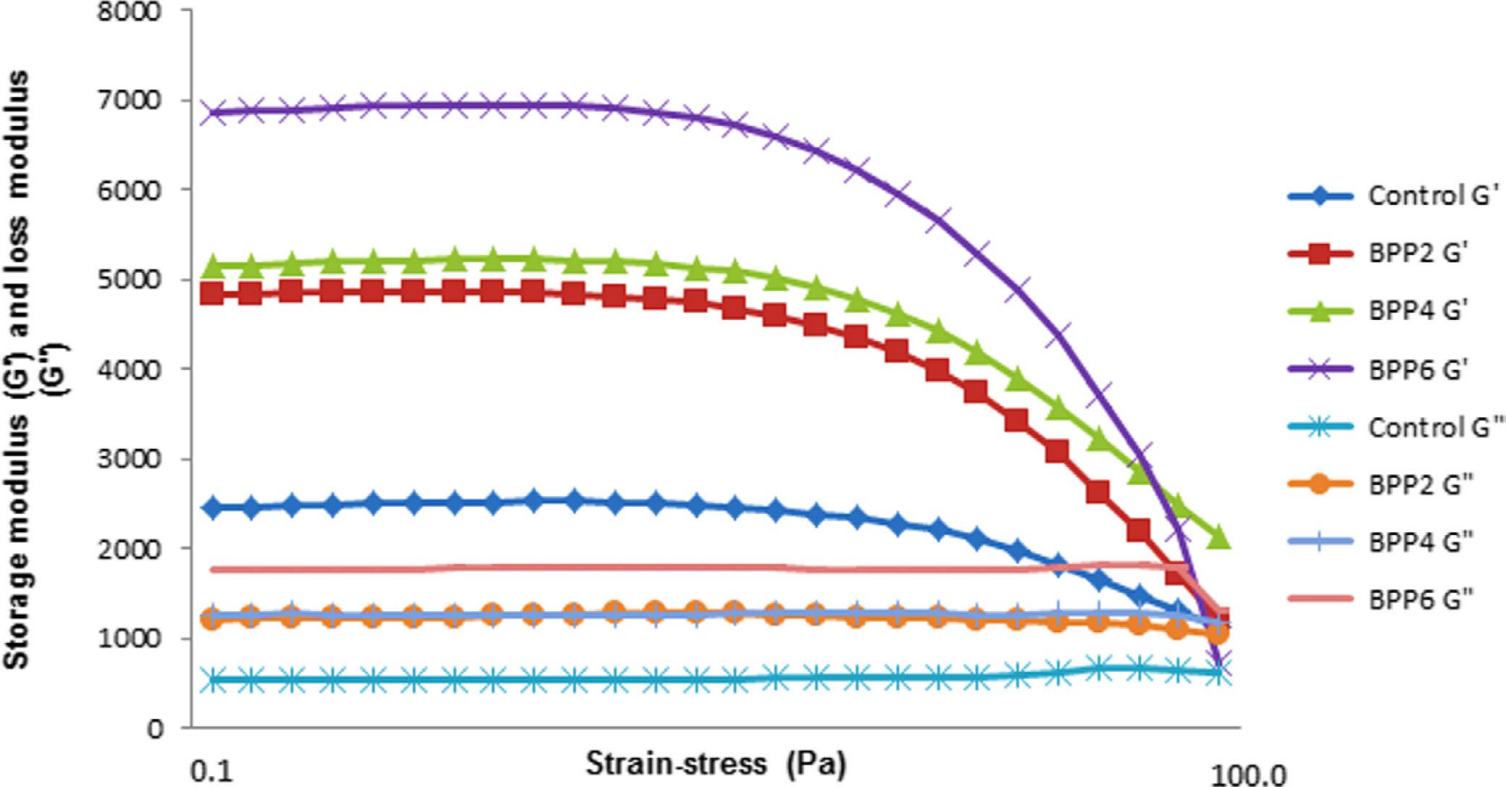
Rheological properties (strain‐sweep test) of chicken sausage with the addition of banana peel powder

Dynamic rheology was used to evaluate the effects of the addition of BPP on the viscoelastic characteristics of chicken sausage (Figure 1). It may be concluded by comparing G’ and G” that storage modulus (G’) was higher compared to the loss modulus (G”), which suggests a gel‐like behavior of the samples. Comparatively, BPP‐containing sausages had a marked increase (*p* < .05) in viscous and elastic moduli, in reference to the control samples, which have a low value of G’ indicating a softer texture compared to the treated samples. With an increase in the BPP added to the system, the value of G’ for meat emulsion increased. The inclusion of BPP could have significantly stabilized the solubilized protein gel and inhibit protein shrinkage because of their filler aspect (Yang et al., 2019). Wanezaki et al. (2015) suggested that the viscous and elastic moduli saw a rise with the addition of fiber to meat products. Results indicated by Hu et al. (2016) also indicate that higher gel elasticity and the network could be due to the emulsified sausage having a substitution of regenerated cellulose. Interestingly, similar pattern of results was observed in the hardness of cooked chicken sausage discussed previously.

Nevertheless, a high level of BPP addition (>2%) caused brittleness and resulted in a significantly lowered linear viscoelastic region (LVR). Viscoelastic region decreased with the increased in BPP's concentration (Figure 1), indicating the decrease in resistance toward applied stress. Samples with high BPP (>2%) tended to shear easily when subjected to high oscillatory stress. This indicates weaker linkages in protein–protein myofibril in high‐BPP samples, which causes breakability. BPP addition at 2% level demonstrated improved protein–protein myofibril linkage and increased resistance against shearing. This also agreed with the findings of texture profiling, where the addition of BPP to the samples reduced cohesiveness, thereby causing brittleness. BPP has a specific solubility level; therefore, it is present in the granular form at higher concentrations. Granular BPP acts as filler and modifies the structure system. Generally, the results of rheology of uncooked chicken sausage (storage modulus and LVR region) and TPA of cooked chicken sausage (hardness and cohesiveness) suggested BPP compromise the microstructural network of chicken sausage. As discussed, the BPP effectively acted as filler which increased the overall textural properties by filling the voids exist in the microstructure of chicken sausage. Yet, the presence of BPP might compromise the microstructural formation by hindering the intermyofibril connection which responsible for cohesiveness or resistance toward stress in meat‐based products. Therefore, these results manifested the role of concentration of added fiber in the overall textural properties, which in case of BPP was at 2% wt/wt.

### 3.6 Sensory profiling

Sensory attributes of chicken sausage formulations were assessed through color, taste, aroma, appearance, juiciness, hardness, and overall acceptability. Table 5 lists the properties of BPP‐containing sausage. BPP2, which refers to a 2% BPP level, caused the hardness attribute to be significantly better compared to that of the control group and those samples having other treatments (*p* < .05). No changes in taste, juiciness, color, or overall acceptability were found between BPP2 and the control group (*p* > .05). The study indicates that the addition of 2%–6% BPP significantly reduced the aroma and appearance of the treated sausage (*p* < .05).

**Table 5:** Sensory evaluation of chicken sausage with different percentage of banana peel powder

	BPP0	BPP2	BPP4	BPP6
Appearance	4.78 ± 1.23^a^	3.91 ± 0.93^b^	3.37 ± 1.29^c^	3.07 ± 1.13^c^
Color	4.46 ± 1.53^ab^	3.91 ± 0.90^b^	2.80 ± 1.52^cd^	2.26 ± 1.20^d^
Aroma	5.21 ± 1.07^a^	4.42 ± 1.06^b^	3.67 ± 1.29^c^	3.32 ± 0.97^c^
Taste	5.15 ± 1.49^a^	4.59 ± 1.31^a^	3.60 ± 1.36^b^	2.59 ± 1.03^c^
Hardness	3.73 ± 1.85^c^	4.67 ± 1.22^ab^	3.62 ± 1.39^c^	2.70 ± 0.96^d^
Juiciness	3.93 ± 1.80^b^	4.09 ± 1.24^b^	3.57 ± 1.63^b^	2.43 ± 1.14^c^
Overall acceptability	4.64 ± 1.19^ab^	4.57 ± 1.02^ab^	3.54 ± 1.52^c^	2.73 ± 0.93^d^

Note

^a‐d^Mean ± *SD* in same column with different superscript indicates that there are significant different (*p* < .05).

Abbreviations: BPP0, control (without banana peels powder); BPP2, added 2 percent banana peel powder; BPP4, added 4 percent banana peels powder; BPP6, added 6 percent banana peel powder.

BPP2 may be accepted by the panelists because of the properties of carbohydrates present in the banana peel powder, which have the ability to retain water and therefore have a better sensory quality in regard to the juiciness and hardness, as indicated by Gravelle, Barbut, & Marangon (2017). Nevertheless, BPP levels over 4% caused a hard texture, and this shows why the overall acceptance declined.

Garcia et al. (2002) suggested that adding fiber‐rich ingredients in foods like biscuit and fermented sausage creates a negative effect on the sensory aspect and product acceptability. The results are in agreement with those of Choi et al. (2011), where the inclusion of 3%–6% of brown rice fiber led to a decrease in the overall acceptability of the sausage.

### 3.7 Lipid oxidation

Quality deterioration, especially secondary lipid oxidation, is a major limiting factor in shelf life of meat products that cause off aromas (Smaoui et al., 2016). Campo et al. (2006) in this study used MDA concentrations around 2 mg/kg as an index of the threshold for oxidized meat acceptability. Table 6 represents the content of TBA during the storage study. In control sausage, lipid oxidation increased drastically (*p* < .05) compared to the sausage containing BPP. The initial TBA value in BPP2 was ranged from 0.1256 to 0.6784. This value was significantly lower than that of the control samples (0.1613–1.2195). In accordance with the study by Pereira & Marschin (2015) and Youryon & Supapvanich (2017), banana peels contain four times more phenolic content than its pulp. This may be contributed to the delaying of oxidation of sausage containing BPP. The study by Someya, Yoshiki, & Okubo (2002) also shows that saba banana peel contains high gallocatechin which has roles as antioxidant, anticarcinogenic, and anti‐inflammation (Machado, Ambrosano, Lage, Abdalla, & Costa, 2017).

**Table 6:** TBA value of sausages with different percentage of banana peel powder stored at 4°C for 30 days

Treatment	Storage time (day)				
	0	7	14	21	30
BPP0	0.1613 ± 0.00^aC^	0.3268 ± 0.00^aC^	0.6510 ± 0.06^aB^	1.1210 ± 0.18^aA^	1.2195 ± 0.15^aA^
BPP2	0.1256 ± 0.00^bD^	0.2641 ± 0.00^bC^	0.3310 ± 0.05^bC^	0.5500 ± 0.04^bB^	0.6784 ± 0.13^bA^

Note

^a‐d^Mean ± *SD* in same column with different superscript indicates that there are significant different (*p* < .05).

^A‐D^Mean ± *SD* in same row with different superscript indicates that there are significant different.

Abbreviations: BPP0, control (without banana peels powder); BPP2, added 2 percent banana peel powder.

## 4 CONCLUSION

This study shows that including BPP caused betterment to water‐holding capacity of chicken sausage, leading to reduced cooking loss. Additionally, BPP improved not only functional aspects like cooking yield or WHC but also has potential health benefits like increase in dietary fiber; however, sensory and textural aspects of the final product were affected. Chicken sausage enriched with dietary fiber prepared using 2% BPP was found to be organoleptically acceptable and textural comparable than the other samples. Therefore, this study establishes the role of concentration of BPP on the technological functionalities of chicken sausage. In other words, though addition of high percentage of BPP improved the nutritional properties, BPP at high concentration compromised the cohesiveness of the chicken sausage. The balance between nutritional properties and textural acceptability is important to sustain the sensorial quality of chicken sausage. For emulsified meat products, BPP optimization and combination with other binding substances require further study. Furthermore, the addition of BPP in sausage had shown delaying of lipid oxidation due to its antioxidant properties. The results demonstrate that banana peel powder (BPP) is a cheap source of dietary fiber. It may be included in meat products because of its benefits in terms of the enhancement of functional characteristics and nutritional composition.

## CONFLICTS OF INTEREST

The authors declare no conflict of interest. The funders had no role in the study design; in the collection, analyses, or data interpretation; in the manuscript writing; or in the decision to publish the findings.
